# Activity in the fronto-parietal network indicates numerical inductive reasoning beyond calculation: An fMRI study combined with a cognitive model

**DOI:** 10.1038/srep25976

**Published:** 2016-05-19

**Authors:** Peipeng Liang, Xiuqin Jia, Niels A. Taatgen, Jelmer P. Borst, Kuncheng Li

**Affiliations:** 1 Department of Radiology, Xuanwu Hospital, Capital Medical University, Beijing 100053, China; 2 Beijing Key Lab of MRI and Brain Informatics, Beijing 100053, China; 3 Institute of Artificial Intelligence, University of Groningen, Nijenborgh 9, 9747 AG Groningen, Netherlands

## Abstract

Numerical inductive reasoning refers to the process of identifying and extrapolating the rule involved in numeric materials. It is associated with calculation, and shares the common activation of the fronto-parietal regions with calculation, which suggests that numerical inductive reasoning may correspond to a general calculation process. However, compared with calculation, rule identification is critical and unique to reasoning. Previous studies have established the central role of the fronto-parietal network for relational integration during rule identification in numerical inductive reasoning. The current question of interest is whether numerical inductive reasoning exclusively corresponds to calculation or operates beyond calculation, and whether it is possible to distinguish between them based on the activity pattern in the fronto-parietal network. To directly address this issue, three types of problems were created: numerical inductive reasoning, calculation, and perceptual judgment. Our results showed that the fronto-parietal network was more active in numerical inductive reasoning which requires more exchanges between intermediate representations and long-term declarative knowledge during rule identification. These results survived even after controlling for the covariates of response time and error rate. A computational cognitive model was developed using the cognitive architecture ACT-R to account for the behavioral results and brain activity in the fronto-parietal network.

The ability to identify environmental regularities is a cognitive skill critical to survival. Human beings have the capacity, called numerical inductive reasoning, to identify and extrapolate numeric rule/patterns involved in numeric materials derived from such diverse areas as scientific discovery, economics, and the weather. Numerical inductive reasoning is associated with calculation, and activity in the fronto-parietal regions of the brain revealed in numerical inductive reasoning^1^,^2^,^3^,^4^,^5^,^6^ was also observed in calculation^7^,^8^. This leads to the suggestion that numerical inductive reasoning (at least when using simple rules) may correspond to a general calculation process rather than necessarily including relation detection and integration (which is critical and unique to reasoning)^9^,^10^. To our knowledge, only one study compared the neural correlates of numerical inductive reasoning with calculation^1^. It was found that compared with calculation, the fronto-parietal regions including the prefrontal gyrus (BA 6), inferior parietal lobule (BA 7, 40) were involved in numerical inductive reasoning. However, in that study it was impossible to exclude the effects of visuospatial processes due to the limitations of the experimental task per se (i.e., numbers located in the reverse triangles). In addition, as no baseline task was included and no behavioral responses were recorded, the difference between numerical inductive reasoning and calculation, as reflected by the fMRI activations, may be confused by other factors such as false activation and task difficulty. Thus, the question whether the neural correlates of numerical inductive reasoning just correspond to calculation or whether numerical inductive reasoning operates beyond calculation remains unclear. In particular, previous studies indicate a critical role of the dorsolateral prefrontal cortex (DLPFC) in numerical inductive reasoning for relation integration and the intraparietal sulcus (IPS) for mental representation^2^,^3^,^4^,^5^. However, it is unknown whether the activation in the fronto-parietal network can distinguish between these processes.

The primary goal of the present study was to directly address these issues. To this end, we ran an fMRI experiment to compare the neural correlates of numerical inductive reasoning (e.g., “7:13:19”) with a calculation task (e.g., “17 − 4 + 5”). It was hypothesized that the fronto-parietal network will be more active in response to numerical inductive reasoning than in response to pure calculation, as the DLPFC will be used for relation detection and the IPS will be used for mental representation during the processes of rule identification. To explain why numerical inductive reasoning would require this additional activity, we built a model in the ACT-R cognitive architecture.

## Cognitive Architecture ACT-R

Adaptive control of thought – rational (ACT-R)^11^ is a cognitive architecture that can provide a description of the processes from input (perception) to output (action) for a wide range of cognitive tasks. It has a computational implementation that can be used to create models of specific tasks, which yield exact predictions in the form of behavioral performance (response time and accuracy) and BOLD response (timing and level of activity).

The ACT-R architecture consists of a set of independent modules that interact through a central production system. For instance, the visual and aural modules process perceptual input, and the manual and vocal modules are used to interact with the world. ACT-R has four central cognitive modules for processing information: the procedural module that implements the central production system, the declarative memory module that manages retrieval requests from declarative memory, the goal module that keeps track of one’s intentions and controls the information processing, and the problem state module (sometimes referred as imaginal module) that maintains intermediate representations necessary for performing a task^11^,^12^. Using this architecture, models of specific tasks can be developed: “ACT-R can be seen as the fixed hardware – the architecture – of the mind, while the models function as software that runs on this hardware”^13^.

The modules of ACT-R have been mapped onto brain regions which are assumed to be active when the corresponding module is active^11^,^13^,^14^. For example, declarative memory retrievals should lead to activity in the prefrontal cortex, the problem state module to the posterior parietal cortex, and visual encoding to the middle occipital cortex^14^. It is not assumed that these are the only regions that are active in response to the modules, and neither that these regions exclusively indicate activity of ACT-R modules. Among these ACT-R modules, two are of particular interest for the current work. The declarative memory module, which is used to store facts and has been mapped onto the DLPFC, is particularly important. Facts in declarative memory have a certain activation level, which determines how easily and how quickly they can be retrieved. The more frequent and the more recent a fact has been used, the easier and faster it is to use it again^11^. The problem state module, which is used to maintain intermediate representations necessary for performing a task, is also important for numerical inductive reasoning. Together these two modules map onto the fronto-parietal network, activity in that network is, according to ACT-R, attributable to the exchange of information between intermediate representations and long-term declarative knowledge.

In order to make fMRI predictions – ACT-R predicts the exact time course of the BOLD response – ACT-R convolves a 0–1 demand function that represents the module activity, *D*(t), with a gamma function that represent the hemodynamic function, *H*(t). If the module is engaged, it will produce a BOLD response *t* time units later according to this gamma function^15^,^16^:


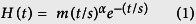


where *m* is the magnitude parameter and determines the height of the function; the parameter *s* is the scale parameter and determines the time scale, and *α* is the shape parameter and determines the narrowness of the function. For a particular module, the cumulative BOLD response can be calculated by convolving the hemodynamic function, *H*(t), with the 0–1 demand function, *D*(t), over the time, which has a value of 1 when the module is active, and 0 otherwise:


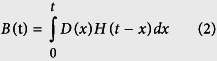


When the timings of buffers actions are all set, we can predict the BOLD functions by estimating the magnitude parameter *m*, the shape parameter *α*, and the latency scale s for each brain region. The second focus of the current study was to employ the computational cognitive model to make specific predictions (in terms of the timing and level of activity) about the different processes of numerical inductive reasoning and calculation.

## Material and Method

### Subjects

Fifteen paid healthy undergraduate and postgraduate students (8 females) with a mean age of 22.1 ± 2.3 years participated in the experiment. All subjects were right-handed and had the normal or corrected-to-normal vision. None of the subjects reported any history of neurological or psychiatric diseases. Written informed consent was obtained from each participant and this study was approved by the Ethics committee of Xuanwu Hospital, Capital Medical University.

### Stimuli

Three types of problems were employed: numerical inductive reasoning (Rea), numerical calculation (Cal), and perceptual judgment (Jud) as a control. The control condition was included to subtract the visual component from the Rea and Cal tasks. All trials were self-paced. We prepared sufficient numbers of each type of problem so that each participant had sufficient trials to do in the limited period of fMRI scanning.

Three numbers separated by colons (for Rea and Jud tasks) or by arithmetic operations (for Cal tasks) were simultaneously presented in the middle of the screen. All numbers were within the range of 1–50 and only addition and subtraction operations (counter-balanced by the two operations) were involved in detecting the rules in Rea and in Cal problems. For Rea problems (“5:11:17”), subjects were required to identify the underlying rule (e.g., “+6”) and to judge whether the sequence formed a valid one (valid if the rules were identical; invalid if not). Within each block of Rea, there was one Rea problem without identical rules (e.g., “19:14:17” with rules of −5 and +3). For Cal tasks (e.g., “13 − 5 + 7”), subjects were required to calculate the answer of the equation (e.g., “15”). The distances between the correct and the incorrect answer options were less than 2 for Cal. For Jud tasks (e.g., “10:44:7”), subjects were required to judge whether there was number “10” in the presented sequence. Half of the Jud task included the number “10” with its positions counter-balanced, and half did not.

### Stimuli presentation

**Figure 1:**
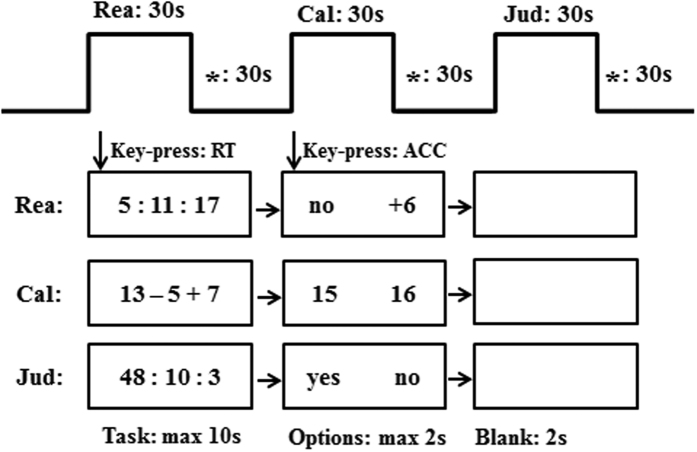
Schematic representation of the fMRI procedure. The three types of problems (Rea, Cal, and Jud) were administered in a run. The order of blocks was randomized with constraint of no more two the same block appeared consecutively.

There were 2 runs in the present study and 9 blocks for each run, with 3 blocks for each condition. Each block lasted 30s followed by 30s fixation for rest. The order of blocks within each run was randomized with the constraint that no more than two of the same blocks appeared consecutively. At the beginning of each block, there was a cue (“Rea” for reasoning problems, “Cal” for calculation problems, or “Jud” for perceptual control) lasting 2s to remind participants what task followed (see Fig. 1 for details). All the tasks were self-paced. If they failed to respond to a problem within 10s, the problem would disappear and a new one was presented. For each task, subjects were required to press left or right button when the answer was acquired and the reaction time (RT) was recorded. After that, two options were displayed, and subjects were instructed to select the answer by pressing the left (if the answer was on the left side) or right (if the answer was on the right side) button. At the same time, accuracy was recorded. Subjects were instructed to respond as accurately and quickly as possible and move to the next trial if the stimuli advanced before they could respond. A 2s blank served as inter-stimulus interval (ISI) and quick responses resulted in more tasks within each block. The response manner was the same for all the tasks.

### MRI data acquisition

Scanning was performed on a 3.0 Tesla MRI system (Siemens Trio Tim; Siemens Medical System, Erlanger, Germany) and with a 12-channel phased array head coil. Foam padding and headphone were used to limit head motion and reduce scanning noise. High-resolution structural images were acquired using a T1 weighted 3D MPRAGE sequence (TR/TE = 1600/2.25 ms, TI = 800 ms, 192 sagittal slices, FOV = 256 mm, 9° flip angle, voxel size = 1 × 1 × 1 mm^3^). Functional images were obtained using an T2* gradient-echo EPI sequence (TR/TE = 2000/31 ms, 90° flip angle, 64 × 64 matrix size in 240 × 240 mm^2^ FOV). Thirty axial slices with a thickness of 4 mm and an inter-slice gap of 0.8 mm were acquired and paralleled to the AC-PC line. The scanner was synchronized with the presentation of every trial. There were a total of 274 fMRI measurements within each run.

### Data preprocessing

Data were analyzed using SPM8 software (http://www.fil.ion.ucl.ac.uk). The first four images for each session were discarded to allow for T1 equilibration effects. The remaining fMRI images were first corrected for within-scan acquisition time differences between slices and then realigned to the first volume to correct for inter-scan head motions (head movement was <2 mm and <2° in all cases). The structural image was co-registered to the mean functional image created from the realigned images using a linear transformation. The transformed structural images were then segmented into gray matter (GM), white matter (WM) and cerebrospinal fluid (CSF) by using a unified segmentation algorithm^17^. The realigned functional volumes were spatially normalized to the Montreal Neurological Institute (MNI) space and re-sampled to 3 mm isotropic voxels using the normalization parameters estimated during unified segmentation. The registration of the functional data to the template was checked for each individual subject. Subsequently, the functional images were spatially smoothed with a Gaussian kernel of 8 × 8 × 8 mm^3^ full width at half maximum (FWHM) to decrease spatial noise.

### fMRI analysis

Each subject’s fMRI series were analyzed into two separate design matrices using a voxel-wise general linear model. At first, block conditions were convolved with a canonical hemodynamic response function (HRF) using a boxcar function lasting the duration of a block. To further exclude the possibility of the contribution of the behavioral performance to the fMRI results, another design with RT and error rate as covariates was conducted.

Condition effects at each voxel were estimated according to the general linear model and regionally specific effects were compared using linear contrast. It produced a statistical parametric map of the *t*-statistic, which was subsequently transformed to a unit normal *Z*-distribution. The contrast images of each subject were then used in a random effect analysis to determine what regions were the most consistently activated across subjects using a one-sample *t*-test.

The contrast of (Rea > Cal), inclusively masking (Rea > Jud) would reveal regions specific to reasoning, and the contrast of (Cal > Rea) inclusively masking (Cal > Jud) would reveal regions specific to calculation. In addition, a conjunction analysis was done to identify brain regions that were commonly activated in Rea and Cal. For the contrasts of (Rea > Cal) and (Cal > Rea), an uncorrected voxel-level intensity threshold of *p* < 0.01 with a minimum cluster size of 25 contiguous voxels was used to correct for multiple comparisons using the AlphaSim method which yielded a corrected *p* < 0.05. For the conjunction analysis, an uncorrected voxel-level intensity threshold of *p* < 0.01 with a minimum cluster size of 150 contiguous voxels was used to correct for multiple comparisons using the AlphaSim method which yielded a corrected *p* < 0.01.

### Regions of interest (ROIs)

To examine the time course of the BOLD signal, we identified functional ROIs based on the contrast of Rea > Cal. All the activated clusters were defined as ROIs. The BOLD responses in these ROIs were computed by taking the average of 7 scans of the rest block (before the onset of task block) and 8 scans of the rest block (after the task block was over) as a baseline. Each point was defined as the percent change from this baseline.

As the area under the curve of the BOLD graph is sensitive to both the magnitude and the duration of the response^12^,^18^, it indicates the total time a module is active and the total activation in a brain area during a trial. We entered these values of the ROIs into an analysis of variance (ANOVA). We only took the area between the start of a task block and the end of the block. This analysis was conducted on both the participants’ data and the model prediction.

### ACT-R modeling

**Figure 2:**
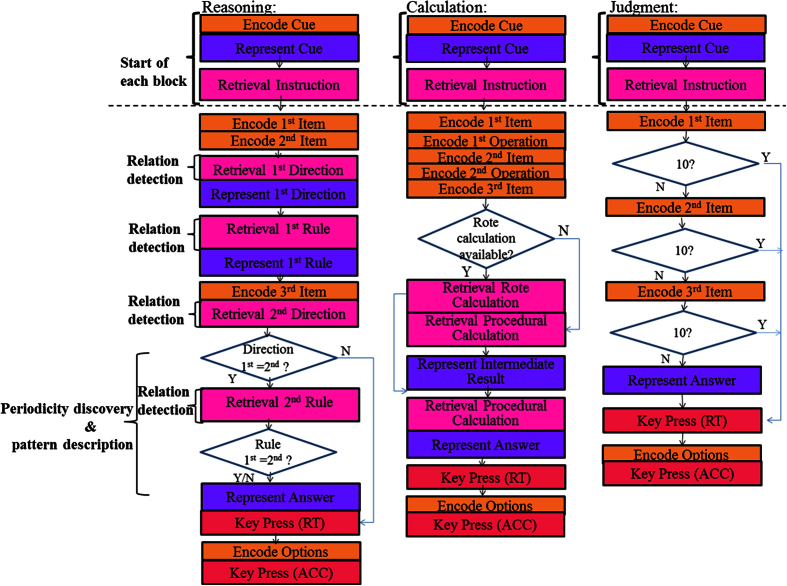
An overview of the model activity in three conditions. Boxes are not drawn to scale but indicate the general pattern of model activity. Orange corresponds to visual activity, blue to problem state, pink to declarative memory, and red-brown to motor activity.

To account for the processes and the behavioral and fMRI data of numerical inductive reasoning and calculation, we developed a computational cognitive model which consists of procedural rules and declarative knowledge in the cognitive architecture ACT-R. Figure 2 shows a schematic of model activity for the three different trial types. In each block condition, the model starts with encoding the cue of the block and represents the information in the problem state module, and begins by retrieving the instruction of the problem. In the reasoning condition, after encoding the first two numbers, the model first quickly determines the direction of the two numbers (i.e., “+” or “−”) by retrieving the order of the numbers from declarative memory and then represents this in the problem state, followed by retrieving the rule/relation between the two numbers and representing it in the problem state. After that, the model encodes the third number, determines the second direction, and compares it with the first direction. If the directions are different, it identifies no regular rule underlying this sequence by approximate calculation. If the two directions are identical, it retrieves the second relation and compares it with first one. It then represents the answer in the problems state module and generates a response. In the calculation condition, after encoding the calculation equation, the model first retrieves the intermediate result by rote memory and represents it in the problem state. It then retrieves the final result by procedural calculation and represents the result in the problem state and generates a response. For the Jud condition, the model encodes the numbers sequentially and represents the answer when the encoding number is “10” or after encoding all the three numbers which are different from “10”, and generates a response.

The model was run 100 times. For the behavioral predictions, there are several parameters that can be estimated, including the time to move visual attention from the screen, to retrieve a declarative memory fact and to modify the contents of the problem state module. All the default values of each parameter could be found in ACT-R 6.0 software (http://act-r.psy.cmu.edu/) by using the command “(sgp)” in LispWorks prompt (www.lispworks.com). For the BOLD prediction, ACT-R predicts the onset and duration of the model component activity for each trial based on the schematic of the model in Fig. 2. For example, the model will start with encoding a stimulus on the screen, followed by a declarative retrieval. ACT-R predicts how long the encoding persists, and when the retrieval starts and how long it takes (the duration of memory retrievals is dependent on the activation of facts in memory). Then, we convolved the activity of the model components with an HRF to enable direct comparison with the BOLD response.

## Results

### Behavioral performance

**Figure 3:**
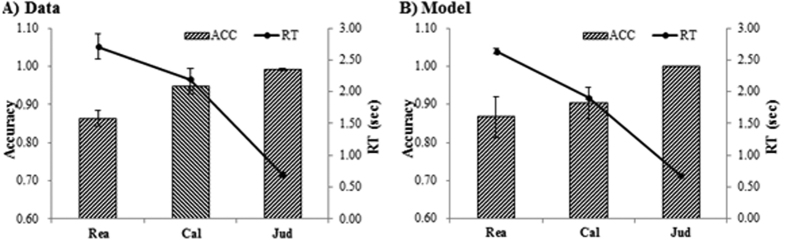
Behavioral performance for Rea, Cal, and Jud problems. (**A**) the data; (**B**) the model fits. Error bars represent the standard error.

The average numbers of trials for the three tasks were 4.96 ± 0.82 (ranging from 3 to 7) for Rea, 5.50 ± 0.82 (ranging from 4 to 7) for Cal, and 8.48 ± 0.67 (ranging from 6 to 9) for Jud. A repeated measures ANOVA showed that response to all three conditions was significantly different both on RT (*F*(2,28) = 83.84, *p* < 0.001) and accuracy (*F*(2,28) = 19.37, *p* < 0.001). In addition, we carried out post hoc comparisons to examine difference between conditions (Fig. 3A). Response to Rea tasks was significantly longer [*F*(1,14) = 12.37, *p* = 0.003; *F*(1,14) = 115.20, *p* < 0.001] and less accurate [*F*(1,14) = 12.72*, p* = 0.003; *F*(1,14) = 35.72, *p* < 0.001] than that of Cal and Jud tasks, respectively. Response to Cal tasks was significantly longer [*F*(1,14) = 102.70*, p* < 0.001] and less accurate [*F*(1,14) = 6.98*, p* = 0.019] than that of Jud tasks.

### fMRI results

**Figure 4:**
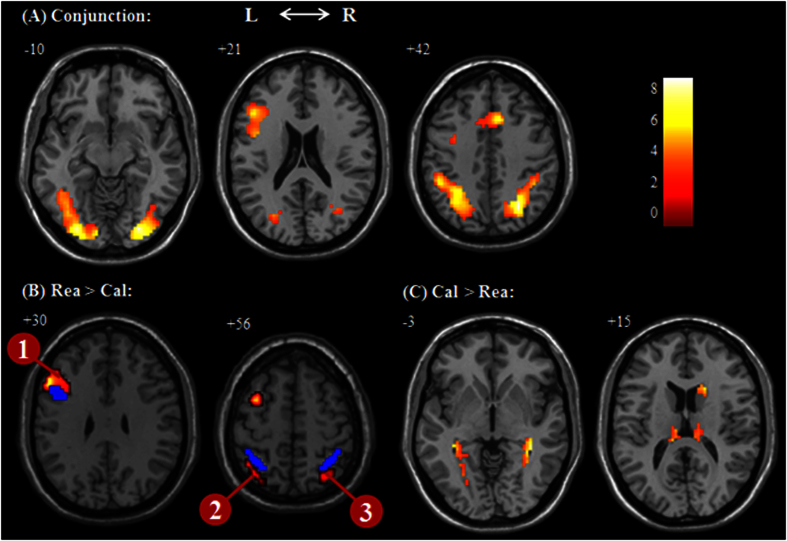
Results of brain activation. (**A**) regions common to Rea and Cal revealed by the conjunction analysis of (Rea > Jud) and (Cal > Jud) using the gray matter as a within mask; (**B**) regions more activated in Rea; (**C**) regions specific to Cal. Number refers to region of interest (ROI); the blue regions refer to the new mapping of ACT-R’ declarative and problem state module (Borst *et al*.^14^).

**Figure 5:**
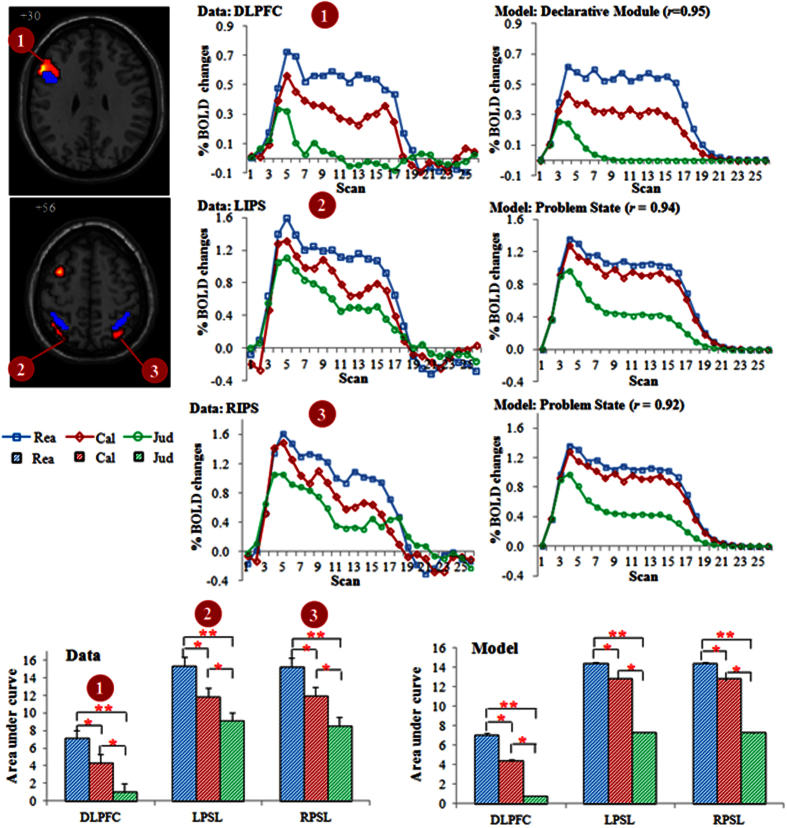
Model predictions of BOLD responses in the defined ROIs. Number refers to the ROI. *refers to *p* < 0.05; **refers to *p* < 0.005.

Conjunction analysis revealed regions commonly activated in Rea and Cal, including the left prefrontal cortex, bilateral anterior cingulate cortex (ACC), intraparietal sulcus extending into the right precuneus, and middle occipital gyrus extending into lingual gyrus and fusiform gyrus (Table 1). Compared with calculation, the regions more specific to numerical inductive reasoning included the left DLPFC and inferior occipital gyrus (BA 18), while the regions uniquely recruited in calculation included the bilateral parahippocampal gyrus, thalamus and right caudate (see Table 2 and Fig. 4). In addition, the bilateral IPS was found more activated for Rea compared with Cal with a relative lower threshold of *p* < 0.05 with a minimum cluster size of 25 contiguous voxels. This finding is confirmed by the ANOVA that showed the area under the curve is significantly higher for Rea than that of Cal in the bilateral IPS (*F*(1,14) = 6.60, *p* < 0.05 and *F*(1,14) = 7.34, *p* < 0.05) (see Fig. 5). Further analysis taking RT and error rates as covariates of no interest, revealed that all the regions survived with the same threshold or with a slightly lower cluster size (see Table S1).

For the ROI analysis, three regions based on the activated clusters, can be grouped (see Table 2) which also survived after controlling the covariates of RT and error rate. The left DLPFC centered at (MNI: −54 21 30) had an overlap with the mapping of ACT-R’s declarative memory retrieval module centered at (MNI: −46, 16, 26)^14^, and the bilateral intraparietal sulcus (IPS) close to the mapping of ACT-R’s problem state centered at (MNI: ±38 −50 48)^14^.

### ACT-R modeling results

The model provides us with latency information of the modules, including visually encoding a stimulus, retrieving an arithmetic fact, representing the information, and generating a response. For the current model all parameters were left at their default values, except for the time it takes to retrieve information from memory (i.e., latency factor) and represents information in the problem state (i.e., problem state change). The two parameters were estimated to be 0.4 for latency factor and 400 ms for the problem state change to fit the model to the behavioral data. The overall fit of the model to the data is reasonably well and the main effect in the data is reflected by the model (Fig. 3B). With the timing parameters set to fit the behavior latencies, the BOLD responses that reflect the retrieval times can be calculated for the prefrontal region. The BOLD response patterns in the DLPFC and bilateral IPS were captured by the model and the model’s predictions for these regions matched the data reasonably well (Fig. 5). As the area under the curve of the BOLD graphs indicates the total activity of a brain region (reflecting both the magnitude and the duration of the response)^12^,^18^, we also computed these values for the three ROIs both for the data and the model (bottom of Fig. 5). The BOLD curves are higher and broader and the brain activities are larger in Rea than Cal and Jud. The ANOVA of the area under the curve confirmed the significantly greater activation for Rea than for Cal both for participants’ data and the model’s prediction (Fig. 5).

## Discussion

This study aimed to examine whether numerical inductive reasoning just corresponds to calculation, or operates beyond calculation, in particular, to explore whether the activation in fronto-parietal network can tell the difference between reasoning and calculation. The result showed that numerical inductive reasoning and calculation share a common brain network including the left prefrontal cortex, bilateral ACC, IPS, and middle occipital gyrus. Compared with calculation, activation in DLPFC and IPS was more specific to numerical inductive reasoning. In addition, regions uniquely activated in calculation were identified in the bilateral parahippocampal gyrus, thalamus, as well as right caudate. These results survived even after controlling for the covariates of response time and error rate. Based on the current experimental tasks (i.e., number series completion) and experimental design, this study could eliminate (at least partly) the potential effect of visual-spatial process (mixed with the rule) and the other factors (including false activation and task difficulty)^1^ on the difference between numerical inductive reasoning and calculation. Additionally, the ACT-R model matched the behavioral data reasonably well and predicted the BOLD responses in the fronto-parietal network which showed more activation in numerical inductive reasoning compared to calculation. The explanation the model offers is that numerical inductive reasoning requires more exchanges in that network.

### Common activations between numerical inductive reasoning and calculation

The findings that numerical inductive reasoning and calculation commonly recruited the fronto-parietal regions are consistent with previous fMRI studies of numerical inductive reasoning^2^,^3^,^4^,^5^,^19^ and mental calculation^7^,^8^. This is also congruent with the cognitive component analysis as reflected in the ACT-R modeling, which indicated that numerical inductive reasoning and calculation share some common processes of manipulating numbers, including encoding the numbers and representing them in problem state, retrieving the answer from declarative memory, and making a response. Further, the common activation in DLPFC may suggest a retrieval effect of arithmetic facts from declarative memory both for the relation detection between numbers for numerical inductive reasoning tasks and the equation calculation for calculation tasks. The recruitment of the bilateral IPS both for numerical inductive reasoning and calculation may be associated with representation of numerical magnitude^20^,^21^,^22^,^23^,^24^. Additionally, the shared activation of ACC in numerical inductive reasoning and calculation (as compared to the control condition) is consistent with its critical role in maintaining the abstract control states that allow cognition to progress correctly^11^, as keeping more track of one’s intentions and controlling the information processing is required both for numerical inductive reasoning and calculation tasks than that of control tasks.

### More activation specific for numerical inductive reasoning: DLPFC and IPS

The greater activation in the DLPFC for numerical inductive reasoning relative to calculation is in agreement with previous studies which have established that the activation of DLPFC is essential for reasoning processes characterized by the requirement of the consideration of multiple relations invoked by reasoning demands^2^,^3^,^4^,^25^,^26^,^27^. Our results are also concordant with several other neuroimaging studies which found that DLPFC is critical for actively building relationships between items during on-line processing^28^,^29^. In addition, IPS has been widely reported to be activated in the numerical inductive reasoning task to represent the intermediate relation between numbers^2^,^3^,^4^.

The finding of more activation in DLPFC and IPS to numerical inductive reasoning was in accordance with our prediction, and we were able to better interpret our results with guidance from cognitive modeling. It should be noted that the ACT-R theory provides a more detailed account of what is behind the retrieval and representational components, which are mapped onto prefrontal and parietal cortices respectively. In the numerical inductive reasoning task, relation detection requires the participant to scan the series and to hypothesize how one element of the series is related to another. The current study relies on two kinds of retrievals: (a) fast retrieval of rote arithmetic memory, and (b) deliberate retrieval when rote memory was unavailable; and two kinds of representations: (a) arithmetic knowledge retrieved during number calculation; and (b) intermediate state of the problem that must be maintained and updated during the processes. Unlike calculation, the solving of numerical inductive reasoning is not routine and not straight-forward, and requires multiple retrievals. As a result, multiple representations are required for numerical inductive reasoning problems, which account for the greater activation in the DLPFC and IPS.

### Regions unique to calculation in the thalamus-caudate regions

In the present study, compared with Rea tasks, one step of one-digit simple calculation is required in Cal problems for which rote memory retrieval is available. The results of such simple one-digit arithmetical problems are stored in declarative memory and usually retrieved directly. The parahippocampal gyrus is known to play a critical role in retrieval of facts form memory^30^. The specific activation of the parahippocampal gyrus in Cal tasks may reflect the greater recruitment of this region to sustain appropriate rote memory representations retrieved from long-term memory. Additionally, according to the triple-code model which describes both the functional architecture and the neural substrates of number processing, when rote memory for a problem is available, the retrieval of simple-arithmetic facts would elicit activity in the left cortico-subcortical loop through basal ganglia and thalamus^31^,^32^. In line with these previous studies, activation of the thalamus and caudate was unique to calculation problems for which rote memory retrieval was available^31^,^33^,^34^. In particular, our model also suggested that participants facilitate their performances by rote memory while solving the calculation problem.

There are still some limitations in this study. First, the current study is a block design, which per se has some insufficiencies. For example, trials with both correct and wrong responses were included in the analysis. Although the accuracies for the three conditions were relative high, a more flexible event-related design was required to replicate the present findings. Second, the task difficulty between the numerical inductive reasoning task and the calculation task was different in this study. There is a possibility that the difference between numerical inductive reasoning and calculation may be mixed by the task difficulty. Therefore, RT and error rate were used as covariates in the current study, which could remove this potential effect to the most extent. However, a new experiment with matched task difficulty between numerical inductive reasoning and calculation is still required in the future to collect more evidences to further test the current findings.

## Conclusions

In general, the present study suggests that with overlapping neural mechanisms sensitive to processes of numerical inductive reasoning and calculation, activity in the fronto-parietal network distinguishes the differential cognitive processes between them. According to ACT-R cognitive architecture, more activity in this network specific for numerical inductive reasoning is attributable to the exchange of information between intermediate representations and long-term declarative knowledge during the processes of rule identification unique to numerical inductive reasoning. The model fits the behavioral results and the BOLD responses in the fronto-parietal network, thus we are able to better understand the underlying differential cognitive processes between numerical inductive reasoning and calculation.

## Additional Information

**How to cite this article**: Liang, P. *et al*. Activity in the fronto-parietal network indicates numerical inductive reasoning beyond calculation: An fMRI study combined with a cognitive model. *Sci. Rep.* **6**, 25976; doi: 10.1038/srep25976 (2016).

## Supplementary Material

Supplementary Information

## Figures and Tables

**Table 1:** Regions activated commonly to Rea and Cal. Lt, left; Rt, right; BA, Broadmann area.

Regions	BA	Cluster size	MNI			*T*-score
			x	y	Z	
Conjunction						
Lt. Inferior/Middle Frontal Gyrus	45/9/46	567	−45	24	24	6.40
Lt. Middle Frontal Gyrus	6		−33	9	60	5.57
Rt. Cingulate Gyrus	32	173	9	18	42	6.59
Lt. Cingulate Gyrus	32		−9	21	36	3.41
Lt. Middle Temporal Gyrus	39	706	−27	−66	27	7.86
Lt. Superior Parietal Lobule	7		−30	−51	45	6.81
Rt. Superior Parietal Lobule	7	516	27	−63	42	8.44
Rt. Precuneus	7		21	−69	51	6.62
Rt. Inferior Parietal Lobule	40		39	−48	51	4.90
Rt. Middle Occipital Gyrus	18	164	27	−84	−9	8.21
Rt. Fusiform Gyrus	37		45	−51	−15	3.44
Lt. Middle Occipital Gyrus	18	349	−30	−87	−6	8.12
Lt. Lingual Gyrus	17		−12	−90	−9	5.90
Lt. Fusiform Gyrus	19		−36	−75	−15	5.83

**Table 2:** Regions specific to Rea and Cal. Lt, left, Rt, right.

Regions	BA	Cluster Size	MNI			*T*-score
			x	y	z	
Rea > Cal						
Lt. Middle Frontal Gyrus①	9	51	−54	21	30	4.96
Lt. Inferior Occipital Gyrus	18	28	−27	−93	−6	3.55
Lt. Inferior Parietal Lobule*②	40	25	−42	−54	60	2.84
Lt. Superior Parietal Lobule*	7		−36	−63	60	2.18
Rt. Superior Parietal Lobule*③	7	25	30	−66	60	3.04
Cal > Rea						
Lt. Parahippocampal Gyrus	19	110	−36	−39	−6	5.74
Lt. Parahippocampal Gyrus	30		−27	−57	3	4.78
Rt. Parahippocampal Gyrus		86	36	−36	−3	6.83
Rt. Parahippocampal Gyrus	30		30	−51	6	5.64
Lt. Thalamus (Pulvinar)		29	−12	−33	12	6.01
Lt. Thalamus			−9	−21	18	5.40
Rt. Caudate (Body)		79	18	21	9	6.17
Rt. Caudate (Head)			18	27	3	5.44
Rt. Thalamus			12	−24	18	4.65

^*^indicates parietal regions activated with a lower threshold of *p* < 0.05. Number within the circle refers to region of interest (ROI). Lt, left; Rt, right; BA, Broadmann area.
