# Supplementary material for: Activity in the fronto-parietal network indicates numerical inductive reasoning beyond calculation: An fMRI study combined with a cognitive model
Source: Sci Rep. 2016 May 19;6:25976. doi: 10.1038/srep25976 (PMC4872123; doi:10.1038/srep25976)
Supplement: Supplementary Information [file srep25976-s1.pdf]

Activity in the fronto-parietal network indicates numerical inductive reasoning beyond calculation: An fMRI study combined with a cognitive model

Peipeng Liang[1,2], Xiuqin Jia[1,2], Niels A. Taatgen[3], Jelmer P. Borst[3], Kuncheng Li[1,2]

[1]Department of Radiology, Xuanwu Hospital, Capital Medical University, Beijing 100053, China
[2] Beijing Key Lab of MRI and Brain Informatics, Beijing 100053, China
[3]Institute of Artificial Intelligence, University of Groningen, Nijenborgh 9, 9747 AG Groningen, Netherlands

Correspondence should be addressed to:
Peipeng Liang
Address: Xuanwu Hospital,
Capital Medical University,
45 Chang Chun Street, Xi Cheng District,
Beijing 100053, China

Date of submission: 2015-07-22

Supplementary material

Table S1. Regions identified by taking RT and error rates as covariates of no interest. * indicates parietal regions activated with a lower threshold of $p < 0.05$. Lt, left; Rt, right.

Regions	BA	Cluster Size	MNI x	MNI y	MNI z	*T*-score
**Conjunction**						
Lt. Inferior Frontal Gyrus	45/46	571	-45	24	24	6.55
Lt. Middle Frontal Gyrus	6		-27	3	51	5.90
Lt. Middle Frontal Gyrus	6		-33	11	55	5.35
Rt. Cingulate Gyrus	32	162	9	18	42	6.33
Lt. Cingulate Gyrus	32		-6	12	45	3.37
Lt. Middle Temporal Gyrus	39	709	-27	-66	27	7.82
Lt. Superior Parietal Lobule	7		-30	-51	45	6.69
Lt. Superior Parietal Lobule	7		-30	-53	39	6.17
Rt. Superior Parietal Lobule	7	536	27	-63	42	8.70
Rt. Precuneus	7		21	-69	51	6.96
Rt. Inferior Parietal Lobule	40		39	-48	51	4.82
Lt. Middle Occipital Gyrus	18	373	-30	-87	-6	8.08
Lt. Fusiform Gyrus	19		-36	-75	-15	5.63
Lt. Lingual Gyrus	17		-12	-87	-6	5.14
Rt. Middle Occipital Gyrus	18	161	27	-84	-9	7.53
**Rea > Cal**						
Lt. Middle Frontal Gyrus	9	57	-54	21	30	4.44
Lt. Middle Frontal Gyrus			-45	24	33	3.40
Lt. Inferior Parietal Lobule*	40	8	-42	-54	60	2.64
Rt. Superior Parietal Lobule*	7	16	33	-63	60	3.12
Rt. Inferior Parietal Lobule*	40	22	42	-51	51	2.89
**Cal > Rea**						
Lt. Parahippocampal Gyrus	30	87	-27	-57	3	5.09
Rt. Parahippocampal Gyrus		107	36	-36	-3	6.88
Rt. Parahippocampal Gyrus	30		30	-51	6	6.02
Lt. Thalamus		29	-12	-33	12	5.95
Lt. Thalamus			-9	-21	18	4.97
Rt. Caudate (Body)		90	12	21	7	7.45
Rt. Thalamus			12	-24	18	5.16
